# The Breast Cancer and the Environment Research Centers: Transdisciplinary Research on the Role of the Environment in Breast Cancer Etiology

**DOI:** 10.1289/ehp.0800120

**Published:** 2009-06-16

**Authors:** Robert A. Hiatt, Sandra Z. Haslam, Janet Osuch

**Affiliations:** 1 Helen Diller Family Comprehensive Cancer Center and; 2 Department of Epidemiology and Biostatistics, University of California at San Francisco, San Francisco, California, USA; 3 Department of Physiology and; 4 Department of Epidemiology, Michigan State University, East Lansing, Michigan, USA

**Keywords:** breast cancer, cohort, environment, etiology, transdisciplinary science

## Abstract

**Objectives:**

We introduce and describe the Breast Cancer and the Environment Research Centers (BCERC), a research network with a transdisciplinary approach to elucidating the role of environmental factors in pubertal development as a window on breast cancer etiology. We describe the organization of four national centers integrated into the BCERC network.

**Data sources:**

Investigators use a common conceptual framework based on multiple levels of biologic, behavioral, and social organization across the life span. The approach connects basic biologic studies with rodent models and tissue culture systems, a coordinated multicenter epidemiologic cohort study of prepubertal girls, and the integration of community members of breast cancer advocates as key members of the research team to comprise the network.

**Data extraction:**

Relevant literature is reviewed that describes current knowledge across levels of organization. Individual research questions and hypotheses in BCERC are driven by gaps in our knowledge that are presented at genetic, metabolic, cellular, individual, and environmental (physical and social) levels.

**Data synthesis:**

As data collection on the cohort, animal experiments, and analyses proceed, results will be synthesized through a transdisciplinary approach.

**Conclusion:**

Center investigators are addressing a large number of specific research questions related to early pubertal onset, which is an established risk factor for breast cancer. BCERC research findings aimed at the primary prevention of breast cancer will be disseminated to the scientific community and to the public by breast cancer advocates, who have been integral members of the research process from its inception.

The Breast Cancer and the Environment Research Centers (BCERC) sponsored by the National Institute for Environmental Health Sciences (NIEHS) and the National Cancer Institute (NCI) were established to better understand how environmental factors may influence pubertal development and to enable primary breast cancer prevention strategies. In 2003, after a period of focused and thoughtful advocacy integrated with scientific consultation, BCERCs were awarded to The Fox Chase Comprehensive Cancer Center in Philadelphia, Pennsylvania; Michigan State University in East Lansing, Michigan; the University of Cincinnati, Ohio; and the University of California San Francisco Helen Diller Family Comprehensive Cancer Center.

In this review, we define the BCERC research questions and present our common conceptual framework for the etiology of breast cancer and the rationale for a focus on puberty. Then, across multiple levels of biologic, behavioral, and social organization, we highlight our current scientific understanding of the development of the normal mammary gland, the nature and action of breast cancer risk factors, and our understanding of the mechanisms of breast carcinogenesis relevant to early development. Our BCERC experimental approach is driven by the knowledge gaps in this understanding. We conclude with a description of the organization of the BCERC.

## Research Questions

We explore whether exposures to environmental factors (e.g., phenols, phthalates, phytoestrogens, genistein, dietary fat, ionizing radiation, psychosocial factors) before and during puberty might set the stage for increased breast cancer risk in adulthood. Multiple hypotheses are being tested by developing and interrogating rodent models and rodent and human cell culture systems to characterize the molecular basis of mammary gland development over the life span. We seek to determine how environmental agents may affect this development and to better understand the process of breast carcinogenesis. In simultaneously conducted epidemiologic cohort studies of prepubertal girls, conceptually integrated with the studies in animal and human tissue models, additional hypotheses posit roles for the environmental, psychological, behavioral, metabolic, and genetic factors as determinants of puberty. This transdisciplinary approach will generate information about relevant exposures from human studies that can be applied to the animal studies. In turn, the animal studies will provide a mechanistic understanding of how environmental exposures may affect pubertal breast development and adversely influence breast cancer risk in adult life.

This research adds to the findings of other similar research relevant to breast cancer, early development, and the environment, including the National Growth and Health Study (Braithwaite et al. 2009), the Child Health and Development Study (Cohn et al. 2003), the CHAMACOS (Center for the Health Assessment of Mothers and Children of Salinas) cohort study (Eskenazi et al. 1999), the Fels Longitudinal Study (Demerath et al. 2007), the Bogalusa Heart Study (Freedman et al. 2002), and the ALSPAC (Avon Longitudinal Study of Pregnancy and Childhood) study in England (Golding 1990), as well as other longitudinal early development studies around the world. The uniqueness of BCERC is the combination of its longitudinal design with questionnaire, biomarker data collected before puberty, the determination of pubertal maturation end points by physical examination, the focus on breast cancer etiology, and the transdisciplinary integration of biologic laboratory-based science, epidemiology, and community participation.

## Methods

### Conceptual Framework: Breast Development and Carcinogenesis across the Life Span

The BCERC initiative is multidisciplinary in its approach to research questions and in the integrated nature of the science with community and advocacy participation. Although not prescribed by the National Institutes of Health’s request for applications, investigators are invested in making our approach truly transdisciplinary (Rosenfield 1992). Transdisciplinary science occurs when scientists from multiple disciplines work interactively on a common problem with a common conceptual model (or framework) and, as a result, develop novel cross-disciplinary methods, insights, and research approaches that would not have occurred with a traditional unidisciplinary investigation. This approach is consistent with the current movement toward team science and interdisciplinary research evidenced by recent reports and funding initiatives [Hiatt and Breen 2008; Stokols et al. 2003; Transdisciplinary Research on Energetics and Cancer (TREC) 2008].

BCERC investigators have agreed on a common conceptual framework that acknowledges the complexity of breast cancer etiologic factors along a life span continuum from pre-natal *in utero* exposures through puberty and maturity to the postmenopausal years. Our framework permits multiple investigators to locate their particular hypotheses within the framework, taking into account our best understanding of pubertal development and the environment from both a socioecological and life span perspective (Smedley and Syme 2001).

At each stage of the life span, the framework recognizes possible influences at multiple levels from genes to cells, tissues, and the organism, through individual behavior, to family, neighborhood, and other upstream social factors.

Although the focus in our study of humans is limited to the prepubertal and pubertal stages of the life span, our animal models allow us to consider the mechanistic basis for the effects of environmental exposures on the mammary gland across the life span. This in turn will provide a bridge for the development and testing of mechanism-based prevention strategies in humans.

### Rationale for Focus on Puberty

The onset of puberty in girls is defined clinically by the first signs of breast development, pubic hair, and other secondary sex characteristics (Grumbach and Styne 2002). The rationale for our focus on puberty stems from the well-established observation that a later onset of menarche is associated with a decreased risk of breast cancer in adult life (Kelsey and Bernstein 1996). The magnitude of the decreased risk is in the range of 9% and 4% for each additional year of menarche delay for pre- and postmenopausal women, respectively (Clavel-Chapelon 2002; Hsieh et al. 1990). The risk for breast cancer among those with an earlier age of menarche is 1.2–2.2 times greater when contrasted with those with later age of menarche (Garland et al. 1998; Titus-Ernstoff et al. 1998).

The onset of menarche has been used in studies of adult women as a marker of pubertal onset, but the relationship between the onset of puberty and menarche has not been constant over secular time (Euling et al. 2008). In the United States, the correlation between onset of puberty and menarche was > 0.9 for women born in the 1930s, 0.5–0.7 for those born in the 1950s, and 0.38–0.39 for those born in the 1970s (Biro et al. 2006). To complicate matters further, a longitudinal study that followed girls annually noted that development of either primary breast areolar or pubic hair, usually breast, lagged behind maturation of the other factor in more than half the cohort (asynchronous maturation) (Biro et al. 2003). These results need to be confirmed in other longitudinal studies (Euling et al. 2008) but suggest that factors contributing to onset of puberty and menarche were more similar in the past than in more recent years, and the clear differentiation is needed between their time of onset and the duration between them (tempo).

It is also well established that both the age at which girls begin puberty and their age of menarche have declined over the last century (Euling et al. 2008). This strongly suggests that changes in lifestyle and/or environmental factors have influenced these trends. Early development is considered an adverse effect because of its impact on outcomes in adolescence, such as accelerated skeletal maturation and short adult height, unwanted pregnancies, sexually transmitted infections, and psychosocial difficulties, and because of its potential impact on adult diseases such as breast cancer and elements of the metabolic syndrome (Golub et al. 2008).

When menarche is accelerated without a concomitant acceleration in the timing of menopause, it is widely thought that the increased duration of hormone exposure over a lifetime promotes the development of breast cancer (de Waard and Thijssen 2005). However, it may be that the pubertal transition itself is critical because of rapid breast development and the susceptibility of rapidly duplicating cells to environmental insults (Berkey et al. 1999). Compared with other times in life, the pubertal breast in the rodent contains the highest number and the greatest proliferative activity of the terminal duct lobular units (TDLUs), the functional units of the breast (Rudland 1993). This may be related to the apparent susceptibility of the breast to carcinogens during puberty (Colditz and Frazier 1995; Knight and Sorensen 2001).

Thus, puberty is a critical period during which to assess the impact of exposures to endocrine disruptors in the environment that interfere with normal hormonal synthesis and metabolic pathways, such as agents in the families of phenols, phthalates, and phytoestrogens (Buck Louis et al. 2008). Endocrine disruptors found in the environment may influence both the timing and pace of the pubertal transition as well as female reproductive outcomes and early-life development (Crain et al. 2008). It is also likely that constitutional factors play a role in age at puberty, and that genetic variability in pathways that influence maturation, such as hormonal and growth factors, may affect onset of puberty and menses. The effects of environmental exposures or lifestyle factors may be most pronounced among those whose genetic or other biologic characteristics make them most susceptible to mediating pathways (Freedman et al. 2002; Pathak et al. 2000).

### Gaps in Knowledge and the BCERC Experimental Approach

Two major hypotheses are being investigated by BCERC: First is that puberty is a window of biological susceptibility to carcinogenesis; second is that the advent of puberty initiates a window of hormonal stimulation ending in menopause. These are not mutually exclusive, and some environmental agents may perturb both. The focus of the biologic studies is on the characteristics of breast development in normal and cancer-prone rodent models, as well as the influence of environmental factors on breast development and breast carcinogenesis. The focus of the epidemiologic studies is on the elucidation of multiple determinants of early puberty with data collected longitudinally over a 5-year period to encompass the first signs of pubertal maturation. The cohort is composed of 1,222 girls recruited with mean ages at baseline of 7.13 years in Cincinnati, 7.34 years in New York City, and 7.38 years in the San Francisco Bay Area. A parent or guardian identified 34.4% of the girls as white non-Hispanic; 25.3% as black non-Hispanic; 4.3% as black Hispanic; 29.9% as Hispanic; 4.5% as Asian; and 1.7% in some other category. Integrated into the structure of the BCERCs are four Community Outreach and Translational Cores (COTCs) that are participants in the research studies and whose members work to translate the current scientific knowledge, as well as findings from new research, into educational messages for the community. Simultaneously, they provide input from the community perspective into the ongoing research of the BCERCs.

#### In utero and prepubertal phases

Although past studies of the association between birth weight and breast cancer risk have yielded inconsistent results, recent meta-analyses have demonstrated an increased risk with larger birth weights (Park et al. 2008; Xue and Michels 2007). This suggests that the *in utero* hormonal milieu may influence breast cancer risk. Trichopoulos and others have hypothesized that higher levels of hormones during pregnancy favor the generation of a higher number of susceptible stem cells with compromised genomic stability (Trichopoulos et al. 2005). Others have posited that *in utero* exposures to estrogens, androgens, insulin-like growth factor 1 (IGF-1), and possibly alpha-fetoprotein might be associated with increased breast cancer risk (Ekbom et al. 1997; Forman et al. 2005).

In animal models, *in utero* stem cells in the fetal breast are capable of cancer initiation, and this may be true in humans as well (Russo and Russo 1996). The presence of a population of stem cells in the fetal breast and their continued presence in the adult reproductive years has been demonstrated in rodent models (Chepko and Smith 1999). The cyclical epithelial proliferation in the breast associated with ovulatory menstrual cycles, as well as the breast’s ability to repopulate itself with each pregnancy is also consistent with the presence of human mammary stem cells (Stingl et al. 2006). These cells, under the influence of hormones and growth factors, may be directly involved in carcinogenesis (Wicha 2008). BCERC investigators are exploring the location and function of stem cells in the mammalian breast to elucidate their possible role in carcinogenesis.

At birth, the breast consists of a rudimentary ductal system. The degree of development at birth is attributable to the influence of maternal hormones (Anbazhagan et al. 1991). By 2 years of age, the breast undergoes involution and is composed of a primitive ductal system without alveoli (Howard and Gusterson 2000). BCERC investigators are seeking a more detailed understanding of the nature of normal mammary development in animal models to provide insights into normal breast development and breast carcinogenesis in humans.

#### Puberty

Until the onset of puberty, the breast remains in a dormant state. At puberty the exact mechanisms responsible for breast development have not been defined, but the initiation of puberty in girls is coincident with the activation of the hypothalamic–pituitary–gonadal (HPG) axis, or thelarche, and the activation of the hypothalamic– pituitary–adrenal (HPA) axis, or adrenarche, which are independent events. A surge of pituitary follicle-stimulating hormone (FSH), associated with HP axis activation, stimulates primordial ovarian follicles to secrete estrogen. Circulating estrogen in turn induces mammary duct, mammary stromal connective tissue, pituitary luteinizing hormone (LH), and vascular proliferation and growth (Rogol 1998). The ratio of FSH to LH favors FSH at the onset of puberty, and even with the onset of menarche, ovarian function continues to be anovulatory (MacMahon et al. 1982b). The activation of the HPA axis stimulates the adrenal production of dehydroepiandrosterone, dehydroepiandrosterone sulfate, and androstenedione, which lead to the development of secondary sexual characteristics including pubic hair, acne, and body odor. The relative timing of thelarche and adrenarche may differentially determine the onset of menarche (Biro et al. 2006). The duration of anovulatory menstrual cycles after the onset of menarche varies from 1 to > 6 years, with longer intervals to ovulation in women with a late menarche (Clavel-Chapelon 2002; MacMahon et al. 1982a). The shorter the period of anovulation, the higher the breast cancer risk (Henderson et al. 1981).

The manifestation of breast development that we are studying in the epidemiologic project is assumed to reflect the underlying mammary biology studied in rodent models in the biology project. In rodent models, we are characterizing the rate of development, extent, and character of the mammary epithelium. With improved animal models and biomarkers to study the impact of environmental stressors on breast cancer, we can elucidate the effects of the timing of these exposures during critical windows of vulnerability.

Focusing now on the phase of pubertal development, we describe factors of influence at multiple levels of organization, starting with genes and concluding with the social environment.

#### Genes

Relatively little is known about how genetic makeup influences breast cancer risk at the time of puberty. It is unclear, for example whether BRCA mutations interact with environmental factor to increase risk (Chang-Claude et al. 2007; Kotsopoulos et al. 2007). Mutations and polymorphisms in genes that control the synthesis and metabolism of hormones or environmental toxicants that act as endocrine disruptors may, however, have some role in pubertal development.

It has been demonstrated, for example, that high-activity *CYP3A4* (cytochrome P450 3A4) alleles, which primarily metabolize testosterone, are associated with early puberty and are more common in African-American than Hispanic or white girls (Kadlubar et al. 2003). There may also be important interactions of these genes with body mass, such that girls with higher body mass indices (BMIs) are more likely to overexpress these genes and enter puberty at earlier ages. BCERC investigators are using new technologies to assess gene and protein expression to elucidate the effects of multiple genetic polymorphisms, including those affecting hormone synthesis and metabolism and carcinogen metabolism. As another example, leptin is important in normal regulation of childhood weight gain, maturation, and development of secondary sexual features. Leptin levels, which are thought to reflect body composition and are best predicted by BMI (Charmandari et al. 2002), rise with age before puberty, suggesting that a threshold effect may trigger puberty (Ong et al. 1999). Polymorphisms in the promoter region of the leptin gene, which have been shown to affect tissue leptin secretion rates (Hoffstedt et al. 2002), could affect age at onset of puberty, particularly in relation to other factors such as BMI, diet, and physical activity. Although analysis of a large data set did not show associations between polymorphisms in the leptin receptor and BMI in adults (Heo et al. 2002), these variants could play a greater role in pubertal development.

Common variants in *LH* and *FSH* genes could also impact timing of puberty (Lamminen and Huhtaniemi 2001). The FSH receptor gene has two distinct isoforms, with ovarian response to FSH stimulation dependent on the *FSHR* genotype (Perez Mayorga et al. 2000). FSH secretion is regulated partly by inhibin, and inhibin B levels correlate positively with age and FSH concentrations several years before the onset of puberty, with concomitant increases during breast development (Crofton et al. 2002).

Genetic variability may also influence age at puberty indirectly, such as genetic predictors of body size. Furthermore, the potential effects of environmental exposures may be mediated by genetic differences in metabolic pathways. The effects of lifestyle factors, such as physical activity, on maturation could be influenced by constitutional factors. In the BCERC, we will investigate the role of genetic polymorphisms that may directly influence maturation or that may interact with environmental and lifestyle factors to impact age at puberty.

#### Metabolic pathways

The exact mechanisms through which underlying hormonally related risk factors work are not known (Aupperlee et al. 2005–2006; Clavel-Chapelon 2002). However, the ovarian hormones, estrogen and progesterone, are believed to play an important role in the etiology of breast cancer (Henderson and Feigelson 2000). A recent systematic review found 15 prospective studies of endogenous hormones and breast cancer risk in postmenopausal women that consistently showed that the highest quintile of estradiol and testosterone conferred an increased relative risk in the range of 2.0–2.2 compared with the lowest quintile (Cummings et al. 2009). In addition, exogenous progestins, used in combination with estrogens in menopausal hormone replacement therapy (HRT), increase breast cancer risk, whereas estrogen-alone HRT is not associated with an increase (Greiser et al. 2005; Rossouw et al. 2002). Thus, to achieve a better understanding of the mechanisms of progesterone action in the breast, BCERC investigators are using *in vivo* and *in vitro* mouse and rat mammary gland models to advance our understanding of the mechanisms of action of the two isoforms of progesterone, PR-A and PR-B, and their specific functions in normal mammary gland development during puberty, at sexual maturity, during and after pregnancy, and after carcinogen exposure in mammary cancer development.

There is also evidence that certain transcription factors such as Stat5, Id2, and C/EBPβ are involved in alveologenesis and are effectors of progesterone and prolactin-driven mammary gland development (Miyoshi et al. 2002; Seagroves et al. 1998). Beyond the potential for dysregulation by steroid disruptors, Stat5 (Paukku and Silvennoinen 2004) and C/EBPβ (Ramji and Foka 2002) can also be activated by proinflammatory cytokines, thus presenting another avenue for environmental influences on mammary gland development being explored by BCERC scientists.

#### Cells and tissues

The cells in the TDLU of the human breast are at their peak of cell replication during puberty and through early adulthood. In animal studies, the susceptibility of the TDLU to neoplastic transformation has been confirmed by *in vitro* studies that have shown that this structure has the highest proliferative activity and rate of carcinogen binding to DNA. More important, when treated with carcinogens *in vitro,* the epithelial cells express phenotypes indicative of cell transformation (Russo et al. 1993). These studies indicate that, in the human breast, the target cell of carcinogens is found in a specific compartment whose characteristics are the determining factors in initiation. These TDLU are believed to contain stem cells that will become the targets of the neoplastic event, depending on *a*) topographic location within the mammary gland tree, *b*) age at exposure to a known or putative genotoxic agent, and *c*) reproductive history of the host. Further, this structure has the highest proliferative activity, estrogen receptor (ER) content, and rate of carcinogen binding to DNA (Russo and Russo 2008).

Epidemiologic studies demonstrate that an early first pregnancy and an increase in parity are associated with a decrease in the risk of breast cancer, each additional live birth conferring a 10% risk reduction (Lambe et al. 1994). Several hypotheses have been put forth to explain the protective effect of early pregnancy: induction of differentiation, decreased proliferative activity, altered hormonal milieu, and alteration in cell fate (Sivaraman and Medina 2002). In both rats and mice, estrogen and progesterone, in the absence of pregnancy, can reproduce the protective effect. Induction of differentiation per se cannot explain the beneficial effect, as treatments with differentiation-inducing hormones or drugs fail to confer the protective effect (Guzman et al. 1999). Likewise, it is unlikely that decreased proliferative activity or reduced cellular levels of ER and PR in parous animals can explain the protective effect of pregnancy (Kariagina et al. 2008; Sivaraman and Medina 2002). An alternative cell-fate hypothesis (Russo et al. 2008; Sivaraman and Medina 2002) proposes that the hormones of pregnancy induce a molecular switch in stem cells that produces cells with persistent changes in regulatory pathways that control proliferation and response to DNA damage. These cells may be able to metabolize carcinogens and repair DNA damage more efficiently than cells of the virginal gland and are thus less susceptible to carcinogenesis. The higher incidence of breast cancer observed in nulliparous women may be the result of the persistence of unmodified stem cells.

Pregnancy also can confer an increased risk of breast cancer for a period of time after delivery (Pathak 2002). Postlactational involution has been shown to cause profound changes in the stromal environment. It has been hypothesized that this stromal environment change may favor the growth of incipient cancers and be the basis for increased risk of breast cancer after pregnancy (Schedin et al. 2007). Understanding the basis of pregnancy-induced protective and/or promotional effects may lead to the development of novel strategies for the prevention of breast cancer.

Mammary epithelial development also depends on molecules produced by the stromal cells (Wiseman and Werb 2002), including fibroblasts, adipocytes (Iyengar et al. 2003), macrophages, eosinophils (Gouon-Evans et al. 2002), and mast cells. Molecules involved in the communication between the microenvironment and the epithelial cells include EGF, FGF2, FGF10, HGF, fibro-blast secreted protein, transforming growth factor β (Bhowmick et al. 2004; Muraoka-Cook et al. 2006), the chemokine CXCL-12, (stromal derived factor 1α), type I collagen (Ingman et al. 2006), matrix metalloproteinase (MMP)-13, MMP-3, and MMP-14 (membrane-type 1 MMP) (Page-McCaw et al. 2007; Sternlicht et al. 2006). Stromal cells also influence steroid hormone responses in the epithelium (Haslam and Woodward 2003). Changes in these stromal cells as the result of environmental stressors may therefore not only perturb normal pubertal development, but create a nurturing environment for a developing mammary neoplasm (Barcellos-Hoff and Ravani 2000; Maffini et al. 2004).

The microenvironment can even prevent malignant cells from committing to neoplasia. Restoration of normal microenvironmental signaling can reverse the malignant phenotype even though the cancer cells retain all of their neoplastically transforming mutations (Bissell and Radisky 2001). In addition, although a normal stroma may protect the epithelium from tumorigenesis, an aberrant stroma can initiate tumorigenesis (Barcellos-Hoff and Ravani 2000; Bhowmick et al. 2004; Maffini et al. 2004). Alterations in stromal composition can also alter steroid hormone responses in cancer cells and affect responses to endocrine therapies (Xie and Haslam 2008). These stromal cells appear to carry on many normal functions, but they drive transformation by hijacking normal cellular responses and inducing them at the wrong place or at the wrong time (Jessani et al. 2004). Further elucidation of the interactions between the epithelial and stromal elements of the developing breast and the susceptibility of these interactions to environmental factors is a key focus for BCERC investigators.

#### Individual behavior and psychosocial factors

Diet and the nutritional content of food consumed in the prepubertal years may be the key factor leading to prepubertal overweight and obesity, which is at the forefront of suspected contributors to early puberty (Kaplowitz 2008). Recent national surveillance data show that 19% of U.S. children 6–11 years of age are overweight as defined by being above the 95th percentile for their age in BMI (Ogden et al. 2006). Although studies have established that girls with higher BMIs are likely to experience puberty at an earlier age (Biro et al. 2003), the mechanisms by which this occurs and the determinants of prepubertal obesity are not well understood (Jasik and Lustig 2008).

Obesity during childhood may be associated with increased risk of premenopausal breast cancer (Weiderpass et al. 2004). Obesity in the prepubertal period may also set the stage for the effects of overweight and obesity in later life. During adulthood, most data suggest that obesity is associated with increased risk of postmenopausal breast cancer, even though it is protective for pre-menopausal breast cancer (Magnusson et al. 2005; Verla-Tebit and Chang-Claude 2005). Obesity represents a constellation of physical attributes that include BMI and waist and hip circumference. This is relevant because in the New York University Women’s Health Study and the EPIC (European Prospective Investigation Into Cancer and Nutrition) Study, premenopausal breast cancer risk was inversely correlated with BMI; however, when adjusted for BMI, waist and hip circumference were associated with increased breast cancer risk (Sonnenschein et al. 1999). Mechanistic explanations as to how obesity may be protective in premenopausal women include decreased estrogen levels in obese women, who have anovulatory cycles (Pike et al. 1993), but the epidemiologic evidence to support this is inconsistent (Garland et al. 1998).

Overall caloric intake, the role of individual nutrients, and dietary intake of substances such as phytoestrogens may also play a role in pubertal development. For example, urinary concentrations of phytoestrogens, particularly daidzein and genistein, have been associated with later age of breast development in girls in New York City, and this protective effect was more pronounced in girls with lower BMIs (Wolff et al. 2008). Further investigation of the effects of these substances is another focus of BCERC investigators.

The impact of decreased physical activity independent of energy intake and its effects in different subgroups of children require further investigation. In the BCERC epidemiologic study, investigators are assessing dietary intake by 24-hr recalls administered to the primary caregiver at four times each year during the prepubertal baseline year. Likewise, detailed physical activity patterns are being assessed. Growth patterns are being closely monitored by annual or biannual assessments of height, weight, anthropometric measures, and body fat measurements.

Psychosocial factors are of interest because they have also been found to influence timing of puberty among girls (Bogaert 2005; Ellis and Garber 2000; Graber et al. 1997). Reproductive maturation appears to accelerate in stressful family contexts, characterized by low-quality parental investment, high levels of stress, negative relationships, and prolonged distress (Romans et al. 2003). In contrast, family relationships characterized by warmth, cohesion, and stability consistently appear to have a protective effect and predict later pubertal onset (Graber et al. 1995). Maternal depression, which is characterized by withdrawal and lack of engagement, has also been linked to early maturation for female children (Ellis and Garber 2000). The proposed mechanism through which acceleration likely occurs is via activation of the stress-mediating HP and sympathoadrenal systems (Grumbach and Styne 2002). Moreover, studies have linked absence of a biological father to early pubertal maturation and indicate that girls growing up in father-absent homes are about twice as likely to experience menarche before 12 years of age (Mustanski et al. 2004; Quinlan 2003). A review of the father-absence literature suggests that menarche among girls in father-absent homes occurs 2–5 months earlier than in homes with father involvement (Ellis 2004), whereas mother absence does not appear to influence pubertal timing (Bogaert 2005). Although more tenuous, childhood psychopathology may also be a significant risk factor for early puberty. A short-term longitudinal study (Hayward et al. 1997) indicated that a cluster of internalizing symptoms, including depression, predicted advanced puberty among girls about 5 months earlier than their same-age peers, and increases in prepubertal anxiety have also predicted earlier pubertal onset, independent of family adversity (Tremblay and Frigon 2005).

In BCERC, to assess the importance of these and other psychological factors, standardized measures are being used longitudinally to evaluate constructs, including child psychopathology [Behavior Assessment System for Children, Parent Report (Reynolds and Kamphaus 2002)], maternal depression [Center for Epidemiologic Studies Depression Scale (Radloff 1977)], family environment [Family Environment Scale (Moos 1990)], and in the Cincinnati Center only, child self-reported depression [Childhood Depression Index (Kovacs 1992)].

#### Environment

Factors at the environmental level can influence pubertal development and ultimately breast cancer in three major ways: *a*) the physical environment—exposure to environmental pollutants or toxicants; *b*) the built environment, which influences whether children have a healthy place to live, play, and go to school; and *c*) the social environment—the associations with socioeconomic status (SES) and race/ethnicity, social norms of behavior, and culture. These are contextual aspects of place as opposed to constitutive aspects, which are the sum of the individual characteristics of persons living in any particular geographically defined area.

##### The physical environment

The impact of putative environmental toxicants on breast development and carcinogenesis is at the heart of BCERC studies. A broad array of environmental carcinogens is being evaluated in animal models and/or in the epidemiologic studies.

Xenoestrogens are part of a large group of synthetic and naturally occurring agents termed endocrine disruptors because of their capacity to perturb normal hormonal actions. It has been suggested that some endocrine disruptors may contribute to the development of hormone-dependent cancers (Sonnenschein and Soto 1998). One ubiquitous source of endocrine-disrupting chemicals is personal care products and cosmetics (Wolff et al. 1996), which may include substances in the categories of parabens, phthalates, and organic solvents. Butyl benzyl phthalate (BBP) is an estrogenic compound and partial agonist for the ER (Zacharewski et al. 1998) widely used in plastic food wraps and other plastics, as well as in cosmetic formulations. Animal studies in rats have shown that prenatal exposure to 500 or 1,000 mg/kg BBP or 250 or 375 mg/kg of its major metabolite, monobenzyl phthalate, induced significant alterations in the reproductive system of male offspring, including undescended testes and decrease in the anogenital distance (Ema and Miyawaki 2002; Ema et al. 2003). Several *in vitro* tests have demonstrated the estrogenic activity of BBP (Hong et al. 2005; Zacharewski et al. 1998), but there is poor evidence on the mechanism mediating the effect of BBP on cell proliferation. It is likely that the estrogenic response is elicited not only via the ER, but also through the activation of other still-unknown pathways (Baker et al. 1999).

The widely used industrial monomer bisphenol A (BPA), another xenoestrogen, is polymerized in the manufacture of polycarbonate plastic and epoxy resins. Human exposure occurs by the leaching of BPA from plastic-lined food and beverage containers and from some dental sealants (Brotons et al. 1995; Olea et al. 1996), and the rate of leaching may greatly increase when the polycarbonate polymer is scratched and discolored (Howdeshell et al. 2003). BPA was found in 95% of urinary samples tested in a large study in the United States (Calafat et al. 2005). Evidence for estrogenic effects of BPA has been reported in several studies showing that it activates ERα and ERβ (Matthews et al. 2001; Routledge et al. 2000) and stimulates MCF-7 breast cancer cell growth (Krishnan et al. 1993). There is some uncertainty as to the level and risk of exposure to BPA in humans, but evidence suggests that it can disrupt normal reproductive tract development in male and female rodents (Ramos et al. 2001; Suzuki et al. 2002).

Persistent organic pollutants (POPs) are a class of chemicals that are lipophilic and resistant to degradation and thus persist in the food chain and individual fat stores. POPs include chemicals such as aldrin, chlordane, DDT (dichlorodiphenyltrichloroethane), dieldrin, heptachlor, and polychlorinated biphenyls (PCBs), which have become highly prevalent in the environment of industrialized countries since World War II. A study of brominated flame retardants (polybrominated biphenyls) among accidentally exposed farm workers in Michigan revealed an association with earlier pubic hair, but not breast development, in the daughters of exposed mothers (Blanck et al. 2000). Polybrominated diphenyl ethers are a subgroup of flame retardants that are now being phased out because of proven toxicity, but a number are still in common use and are under study in relationship to pubertal development. Pesticides, which are ubiquitous in the environment and can be measured in biospecimens from all U.S. adults and children, are POPs associated with earlier menarche in several (Gladen et al. 2000; Ouyang et al. 2005; Vasiliu et al. 2004) but not all studies (Denham et al. 2005). A recent study made use of long-term human data and found that girls who had been exposed to DDT before 14 years of age had a higher incidence of breast cancer than those who had not been exposed (Cohn et al. 2007). PCBs have been inconsistently related to breast cancer in adult women in observational studies, but there is evidence from at least four studies of a gene–environment interaction with *CYP1A1* such that high levels of PCB exposure and expression of *CYP1A1* confer a higher risk of breast cancer (Brody et al. 2007). In another possible gene–environment interaction, certain POPs may interact with *CYP3A4*, a critical enzyme for xenobiotic metabolism as well for endogenous/exogenous hormone metabolism that inhibits the metabolism of endogenous estradiol, thereby potentially increasing serum levels and increasing breast cancer risk through this mechanism (Hodgson and Rose 2007)

Heavy metals such as cadmium and lead are both known or probable carcinogens and also have estrogenic properties (Choe et al. 2003). Lead exposure has been associated with later pubertal onset or menarche in several studies (Denham et al. 2005; Selevan et al. 2003; Wu et al. 2003), but both lead and cadmium have been associated with increased breast cancer risk in human epidemiologic studies (Cantor et al. 1995; McElroy et al. 2006). Environmental tobacco smoke (ETS) has been assessed in girls and is associated with early menarche (Reynolds et al. 2004; Windham et al. 2004). However, mechanisms by which ETS might contribute to earlier menarche remain to be elucidated, especially by examining possible gene–environment interactions such as those with *NAT2* slow acetylator and *GSTMI* null genotypes that may be associated with breast cancer in adults (Ambrosone et al. 2008).

Polycyclic aromatic hydrocarbons (PAHs) are a large class of chemicals formed by the incomplete combustion of coal, oil, and gas, as well as tobacco smoke and other substances to which humans are exposed in ambient air. These substances are genotoxic and known to be potential breast carcinogens (Morris and Seifter 1992), perhaps by damaging DNA through oxidative stress. Animal experiments indicate that puberty and early development may be the period during which the breast is most sensitive to these effects (Fenton 2006). In addition, because air pollution can vary by neighborhood environments, there may be an interaction between PAH exposure and the social environment (Morello-Frosch and Jesdale 2006).

One of the most commonly voiced cancer-related public concerns is the possible impact of hormones in food, because U.S. animal food sources are frequently exposed to growth hormones to boost production of meat, dairy products, and eggs. Because higher levels of estradiol may induce the hypothalamic burst of gonadotropin that initiates puberty, the impact of hormone-treated cattle deserves further study (Massart et al. 2006). In the BCERC studies, questions are being directed to the consumption of organic food consumption as an indirect measure of such exposures.

Finally, among environmental factors, ionizing radiation exposure has long been recognized as a risk factor for breast cancer in humans, probably as a result of the induction of DNA double-strand breaks. An increased risk of breast cancer has been consistently reported for radiation exposure from various sources, including the atomic weapon explosions in Nagasaki-Hiroshima (Tokunaga et al. 1991), and medical treatments for a large number of conditions (Boice et al. 1991; John and Kelsey 1993; Shore et al. 1993), and among radiologic technologists (Boice et al. 1995). Among atomic bomb survivors, increased risk has been related clearly to younger age at exposure (Land et al. 2003). Elevated breast cancer risk in areas with relatively lose doses of radiation contamination from the Chernobyl accident has been noted in Belarus (average cumulative dose ≥ 40 mSv) about 10 years after the incident, and risk was greater among women younger at exposure (Pukkala et al. 2006). BCERC investigators are using radiation as a prototypical breast carcinogen to evaluate genetic and molecular mechanisms of carcinogenesis in rodent models, and we are assessing exposure by questionnaire in the epidemiology study.

For even the partial list of putative environmental agents presented above, much remains to be elucidated, not only in terms of their biologic effects in animal models, but also the risks for adverse outcomes as assessed by human epidemiology. Furthermore, it is likely that chemicals interact with each other and that their individual effects are modified by the presence of genetic polymorphisms and by the social context in which the exposure plays out. It is only with a prospective, longitudinal study with input from multiple disciplinary perspectives, such as in the BCERC, that these effects and interactions can be illuminated.

##### The built environment

In addition to chemical toxicants in the physical environment, there are also aspects of the built environment that can lead to childhood obesity and may contribute to earlier puberty and breast cancer incidence later in life.

For example, it is known that people who live in socioeconomically deprived neighborhoods are more likely to be physically inactive (Cubbin et al. 2006; Yen and Kaplan 1998), to have less healthy dietary habits (Lee and Cubbin 2002), and to be obese (Cubbin et al. 2006). Socioeconomically deprived areas tend to have fewer food stores, more fast food stores, and more liquor stores (Morland et al. 2002). In addition, economic and social measures and other macro-level elements, such as urban sprawl, have been associated with higher rates of obesity (Ewing et al. 2003). The urban design, planning, and transportation literatures show that population density, connectivity, and land-use mix are related in many studies to higher rates of walking and cycling for utilitarian purposes (Saelens et al. 2003). These factors may, in turn, be influenced by existing policies on land use, zoning, and other factors that impact the built environment. BCERC investigators will explore the relationship of the built environment as determined both from interviews with caregivers as well as on-the-ground audits, in the Bay Area Center, of the characteristics of neighborhoods where the girls live. The audit observations will be linked to individual data from longitudinally obtained interviews with the girls.

##### The social environment

Childhood obesity is more prevalent in nonwhite and low-income children, and thus factors associated with SES, race, and ethnicity could be contributing to observed disparities in the onset of puberty. However, the relationship of SES and race/ethnicity to breast cancer is complex. Breast cancer is one of the few cancers related directly to higher SES and to being self-identified as white compared with black (above the age of about 40 years), Hispanic, and Asian women. Within race/ethnic groups, there is also a direct relationship with SES (Kelsey and Bernstein 1996). The most accepted explanation for the direct positive relationship with SES is that higher-SES women tend to have their first child later in life, have fewer children, and have menopause later, all of which raise the risk of breast cancer.

Until recently, the age of menarche has been lower in higher-SES populations and in more industrialized countries. In the last half-century, in the United States at least, this relationship may have changed. Epidemiologic evidence now suggests that lower SES is related to earlier puberty, as determined by entry into stage 2 breast development among girls in the United States (Ellis and Essex 2007). In one multiethnic cohort study, higher maternal education predicted later menarche, but income was unrelated (Windham et al. 2004). The educational dimension of parental SES has also been associated with earlier pubertal development in other studies of predominantly white girls (Davison et al. 2003; Ellis and Essex 2007; Lee et al. 2007).

Although pathways linking SES and menarche activation are not well understood, it appears that body composition and nutrition are essential parts of the puzzle (Lawson 1999). These nutritional factors are likely to affect the endocrine milieu controlled by the HPG system, particularly endogenous estradiol and lower sex hormone–binding globulin (Vihko and Apter 1984). Multiple markers of social environment can alter these hormonal profiles at the time of mammary development and may explain disparity in menarchal age between black and white girls (McClintock et al. 2005). Because distributions of genetic polymorphisms vary by race/ethnicity, the influence of constitutional factors may also influence differences in age at puberty.

## BCERC Organizational Structure

In addition to the four BCERCs described above, a coordinating center at the University of California, San Francisco (UCSF) site exists to coordinate questionnaire development, data entry, centralized data management, and pooled analyses. Bioinformatics support is being coordinated at the Fox Chase Cancer Center (Philadelphia, PA). Other aspects being coordinated between centers include an Internet web site (http://www.bcerc.org), an intranet web site, and national meetings. A publications committee has developed and monitors procedures for the analysis of pooled data, publication tracking, authorship protocols, ancillary studies, and other matters pertaining to pooled data and publications from the network.

Among the three centers that participate in the epidemiologic cohort study, UCSF (Kaiser Permanente), University of Cincinnati, and Fox Chase (Mount Sinai School of Medicine), there is a high degree of collaboration and standardization in the epidemiologic studies across centers to maximize opportunities for pooled analyses (i.e., increased sample size) and cross-site comparisons.

Integrated into the structure of the BCERCs are four COTCs. Members of the COTC include breast cancer advocates as well as academicians with expertise in communication. The primary role of the COTCs is the translation and dissemination of major research findings to the public through their member organizations. Specifically, the COTCs conduct their own research activities in communication and dissemination, develop fact sheets and other educational materials based on BCERC research, hold town hall meetings and other community activities, and assist with recruitment and retention strategies for the epidemiologic study. Members participate on both the epidemiology and biology projects, disseminate educational information and scientific findings of the BCERC and ensure the inclusion of the community perspective in BCERC projects and facilitating the ongoing flow of information from the study team to the community and from the community back to the study team.

Specific results of constituent studies are being published from individual centers and collaborative efforts across centers. The reader is referred to the study Web site (www.bcerc.org) for a listing of these publications to date (BCERC 2008).

## Conclusion

The transdisciplinary nature of our approach derives first from the highly varied areas of expertise of the scientific team and the community partners. The disciplines represented include genetics, molecular and cell biology, immunology, anatomy, radiation biology, pediatrics, endocrinology, toxicology, nutritional sciences, communication science, and epidemiology. The BCERC investigators within the network are addressing specific research questions that focus on the common shared goal of the overall project: to understand the role of the environment in pubertal development as a window on breast cancer etiology. The ongoing and integrated involvement of community members and advocates in the research process and in communication with the public is a unique and effective way to advance the transdisciplinary science approach we have adopted.
